# Supplementary material for: Modeling glioblastoma heterogeneity as a dynamic network of cell states
Source: Mol Syst Biol. 2021 Sep 16;17(9):e10105. doi: 10.15252/msb.202010105 (PMC8444284; doi:10.15252/msb.202010105)
Supplement: Supplementary file 6 — Source Data for Figure 5 [file MSB-17-e10105-s004.zip › Figure5A_sourcedata/GSEA_3071/hallmarks_stateV.GseaPreranked.1623742546329/gsea_report_for_na_neg_1623742546329.html]

Report for na\_neg 1623742546329 [GSEA]

| GS  follow link to MSigDB | GS DETAILS | SIZE | ES | NES | NOM p-val | FDR q-val | FWER p-val | RANK AT MAX | LEADING EDGE || 1 | HALLMARK\_EPITHELIAL\_MESENCHYMAL\_TRANSITION | Details ... | 131 | -0.64 | -3.78 | 0.000 | 0.000 | 0.000 | 513 | tags=47%, list=10%, signal=51% |
| 2 | HALLMARK\_UV\_RESPONSE\_DN | Details ... | 81 | -0.53 | -2.86 | 0.000 | 0.000 | 0.000 | 319 | tags=33%, list=6%, signal=35% |
| 3 | HALLMARK\_TNFA\_SIGNALING\_VIA\_NFKB | Details ... | 91 | -0.49 | -2.69 | 0.000 | 0.000 | 0.000 | 541 | tags=38%, list=10%, signal=42% |
| 4 | HALLMARK\_HYPOXIA | Details ... | 97 | -0.48 | -2.68 | 0.000 | 0.000 | 0.000 | 624 | tags=40%, list=12%, signal=45% |
| 5 | HALLMARK\_COAGULATION | Details ... | 49 | -0.55 | -2.65 | 0.000 | 0.000 | 0.000 | 519 | tags=37%, list=10%, signal=40% |
| 6 | HALLMARK\_IL2\_STAT5\_SIGNALING | Details ... | 81 | -0.49 | -2.63 | 0.000 | 0.000 | 0.000 | 608 | tags=37%, list=11%, signal=41% |
| 7 | HALLMARK\_ANGIOGENESIS | Details ... | 20 | -0.68 | -2.58 | 0.000 | 0.000 | 0.000 | 299 | tags=45%, list=6%, signal=48% |
| 8 | HALLMARK\_INFLAMMATORY\_RESPONSE | Details ... | 56 | -0.52 | -2.57 | 0.000 | 0.000 | 0.000 | 533 | tags=43%, list=10%, signal=47% |
| 9 | HALLMARK\_INTERFERON\_GAMMA\_RESPONSE | Details ... | 66 | -0.50 | -2.56 | 0.000 | 0.000 | 0.000 | 1167 | tags=56%, list=22%, signal=71% |
| 10 | HALLMARK\_ALLOGRAFT\_REJECTION | Details ... | 51 | -0.53 | -2.54 | 0.000 | 0.000 | 0.000 | 1472 | tags=71%, list=28%, signal=97% |
| 11 | HALLMARK\_MYOGENESIS | Details ... | 71 | -0.45 | -2.29 | 0.000 | 0.000 | 0.000 | 475 | tags=28%, list=9%, signal=31% |
| 12 | HALLMARK\_APICAL\_JUNCTION | Details ... | 89 | -0.42 | -2.28 | 0.000 | 0.000 | 0.000 | 1038 | tags=44%, list=20%, signal=54% |
| 13 | HALLMARK\_COMPLEMENT | Details ... | 75 | -0.43 | -2.26 | 0.000 | 0.000 | 0.000 | 794 | tags=36%, list=15%, signal=42% |
| 14 | HALLMARK\_REACTIVE\_OXYGEN\_SPECIES\_PATHWAY | Details ... | 24 | -0.58 | -2.21 | 0.000 | 0.000 | 0.000 | 563 | tags=50%, list=11%, signal=56% |
| 15 | HALLMARK\_INTERFERON\_ALPHA\_RESPONSE | Details ... | 34 | -0.49 | -2.16 | 0.000 | 0.000 | 0.002 | 979 | tags=50%, list=18%, signal=61% |
| 16 | HALLMARK\_ADIPOGENESIS | Details ... | 77 | -0.39 | -2.12 | 0.000 | 0.001 | 0.004 | 1068 | tags=42%, list=20%, signal=51% |
| 17 | HALLMARK\_P53\_PATHWAY | Details ... | 97 | -0.38 | -2.11 | 0.000 | 0.000 | 0.004 | 505 | tags=27%, list=10%, signal=29% |
| 18 | HALLMARK\_CHOLESTEROL\_HOMEOSTASIS | Details ... | 32 | -0.48 | -2.09 | 0.000 | 0.001 | 0.005 | 904 | tags=50%, list=17%, signal=60% |
| 19 | HALLMARK\_UNFOLDED\_PROTEIN\_RESPONSE | Details ... | 51 | -0.42 | -1.99 | 0.000 | 0.002 | 0.022 | 1115 | tags=45%, list=21%, signal=57% |
| 20 | HALLMARK\_MTORC1\_SIGNALING | Details ... | 101 | -0.34 | -1.94 | 0.000 | 0.004 | 0.036 | 1225 | tags=50%, list=23%, signal=63% |
| 21 | HALLMARK\_GLYCOLYSIS |  | 91 | -0.34 | -1.87 | 0.005 | 0.005 | 0.054 | 608 | tags=29%, list=11%, signal=32% |
| 22 | HALLMARK\_HEDGEHOG\_SIGNALING |  | 27 | -0.45 | -1.85 | 0.011 | 0.006 | 0.065 | 532 | tags=33%, list=10%, signal=37% |
| 23 | HALLMARK\_PROTEIN\_SECRETION |  | 37 | -0.41 | -1.82 | 0.002 | 0.008 | 0.090 | 1315 | tags=49%, list=25%, signal=64% |
| 24 | HALLMARK\_KRAS\_SIGNALING\_UP |  | 76 | -0.34 | -1.82 | 0.000 | 0.007 | 0.091 | 475 | tags=24%, list=9%, signal=26% |
| 25 | HALLMARK\_XENOBIOTIC\_METABOLISM |  | 51 | -0.37 | -1.75 | 0.002 | 0.011 | 0.142 | 1277 | tags=51%, list=24%, signal=67% |
| 26 | HALLMARK\_ESTROGEN\_RESPONSE\_EARLY |  | 80 | -0.33 | -1.74 | 0.000 | 0.012 | 0.151 | 625 | tags=25%, list=12%, signal=28% |
| 27 | HALLMARK\_APOPTOSIS |  | 82 | -0.32 | -1.68 | 0.012 | 0.018 | 0.221 | 327 | tags=20%, list=6%, signal=20% |
| 28 | HALLMARK\_WNT\_BETA\_CATENIN\_SIGNALING |  | 16 | -0.47 | -1.62 | 0.038 | 0.027 | 0.323 | 527 | tags=25%, list=10%, signal=28% |
| 29 | HALLMARK\_TGF\_BETA\_SIGNALING |  | 23 | -0.39 | -1.51 | 0.080 | 0.050 | 0.534 | 416 | tags=26%, list=8%, signal=28% |
| 30 | HALLMARK\_ESTROGEN\_RESPONSE\_LATE |  | 77 | -0.27 | -1.46 | 0.033 | 0.069 | 0.659 | 330 | tags=17%, list=6%, signal=18% |
| 31 | HALLMARK\_ANDROGEN\_RESPONSE |  | 50 | -0.27 | -1.30 | 0.124 | 0.154 | 0.923 | 761 | tags=28%, list=14%, signal=32% |
| 32 | HALLMARK\_BILE\_ACID\_METABOLISM |  | 23 | -0.31 | -1.17 | 0.244 | 0.277 | 0.992 | 1004 | tags=35%, list=19%, signal=43% |
| 33 | HALLMARK\_HEME\_METABOLISM |  | 61 | -0.24 | -1.17 | 0.231 | 0.274 | 0.994 | 624 | tags=21%, list=12%, signal=24% |
| 34 | HALLMARK\_IL6\_JAK\_STAT3\_SIGNALING |  | 20 | -0.30 | -1.13 | 0.307 | 0.316 | 0.999 | 1162 | tags=40%, list=22%, signal=51% |
| 35 | HALLMARK\_FATTY\_ACID\_METABOLISM |  | 49 | -0.23 | -1.08 | 0.347 | 0.379 | 1.000 | 513 | tags=18%, list=10%, signal=20% |
| 36 | HALLMARK\_PI3K\_AKT\_MTOR\_SIGNALING |  | 36 | -0.24 | -1.05 | 0.393 | 0.407 | 1.000 | 558 | tags=22%, list=11%, signal=25% |
| 37 | HALLMARK\_MYC\_TARGETS\_V2 |  | 33 | -0.22 | -0.97 | 0.521 | 0.534 | 1.000 | 1932 | tags=67%, list=36%, signal=104% |
| 38 | HALLMARK\_OXIDATIVE\_PHOSPHORYLATION |  | 71 | -0.19 | -0.96 | 0.552 | 0.533 | 1.000 | 2512 | tags=75%, list=47%, signal=140% |
| 39 | HALLMARK\_PEROXISOME |  | 31 | -0.15 | -0.62 | 0.916 | 0.947 | 1.000 | 716 | tags=19%, list=14%, signal=22% |
Table: Gene sets enriched in phenotype **na **[plain text format]****
